# *In Vitro* Activity of Ceftibuten/VNRX-5236 against Urinary Tract Infection Isolates of Antimicrobial-Resistant *Enterobacterales*

**DOI:** 10.1128/AAC.01304-21

**Published:** 2022-01-18

**Authors:** James A. Karlowsky, Meredith A. Hackel, Daniel F. Sahm

**Affiliations:** a IHMA, Schaumburg, Illinois, USA; b Department of Medical Microbiology and Infectious Diseases, Max Rady College of Medicine, University of Manitoba, Winnipeg, Manitoba, Canada

**Keywords:** VNRX-5236, VNRX-7145, ceftibuten, urinary tract infection, oral therapy, *Enterobacterales*

## Abstract

Ceftibuten/VNRX-7145 is a cephalosporin/boronate β-lactamase inhibitor combination under development as an oral treatment for complicated urinary tract infections caused by *Enterobacterales* producing serine β-lactamases (Ambler class A, C, and D). *In vivo*, VNRX-7145 (VNRX-5236 etzadroxil) is cleaved to the active inhibitor, VNRX-5236. We assessed the *in vitro* activity of ceftibuten/VNRX-5236 against 1,066 urinary isolates of *Enterobacterales* from a 2014–2016 global culture collection. Each isolate tested was preselected to possess a multidrug-resistant (MDR) phenotype that included nonsusceptibility to amoxicillin-clavulanate and resistance to levofloxacin. MICs were determined by CLSI broth microdilution. VNRX-5236 was tested at a fixed concentration of 4 μg/ml. Ceftibuten/VNRX-5236 inhibited 90% of all isolates tested (MIC_90_) at 2 μg/ml; MIC_90_s for ESBL- (*n *= 566), serine carbapenemase- (*n *= 116), and acquired AmpC-positive (*n *= 58) isolate subsets were ≤0.25, >32, and 8 μg/ml, respectively. At concentrations of ≤1, ≤2, and ≤4 μg/ml, ceftibuten/VNRX-5236 inhibited 89.1, 91.7, and 93.1% of all isolates tested; 96.5, 97.7, and 98.4% of ESBL-positive isolates; 75.9, 81.9, and 81.9% of serine carbapenemase-positive isolates; and 70.7, 81.0, and 87.9% of acquired AmpC-positive isolates. Ceftibuten/VNRX-5236 at concentrations of ≤1, ≤2, and ≤4 μg/ml inhibited 85-89, 89-91, and 91-92% of isolates that were not susceptible (defined by CLSI and EUCAST breakpoint criteria) to nitrofurantoin, trimethoprim-sulfamethoxazole, and/or fosfomycin, (as part of their MDR phenotype), oral agents commonly prescribed to treat uncomplicated urinary tract infections. The potency of ceftibuten/VNRX-5236 (MIC_90_, 2 μg/ml) was similar (within one doubling-dilution) to intravenous-only agents ceftazidime-avibactam (MIC_90_ 2 μg/ml) and meropenem-vaborbactam (MIC_90_ 1 μg/ml). Continued investigation of ceftibuten/VNRX-5236 is warranted.

## INTRODUCTION

*Enterobacterales* are important etiologic agents of uncomplicated and complicated urinary tract infections. β-lactams are widely prescribed to treat urinary tract and other infections in patients in both community and hospital settings because of their reliable antibacterial activity and favorable safety profile. Unfortunately, the efficacy of β-lactams against Gram-negative pathogens is continuously threatened by the emergence and spread of new β-lactamases (ESBLs, AmpCs and carbapenemases) (1–3). A proven strategy to address the treatment challenges associated with evolving and proliferating β-lactamases involves the development of new agents that combine a novel β-lactamase inhibitor with an approved β-lactam to prevent its hydrolysis (4, 5). In the last decade, several new parenteral β-lactam-β-lactamase inhibitor combinations have been introduced into the clinical use (ceftazidime-avibactam, imipenem-relebactam, meropenem-vaborbactam and ceftolozane-tazobactam) (4, 5). In contrast, a new orally bioavailable β-lactam-β-lactamase inhibitor combination has not been approved since amoxicillin-clavulanate in the 1980s. Today, resistance to amoxicillin-clavulanate among *Enterobacterales* is a major concern, because increasing numbers of clinical isolates carry ESBLs as well as KPC and OXA-48-like carbapenemases. These enzymes were not a clinical concern when amoxicillin-clavulanate was first introduced. Clavulanic acid possesses inhibitory activity against only certain Ambler class A ESBLs but demonstrates essentially no activity against class C cephalosporinases (AmpCs) or class A (KPC) or class D (OXA-48-like) carbapenem hydrolyzing enzymes. New oral antimicrobial agents to treat outpatients with urinary tract infections caused by *Enterobacterales* carrying serine β-lactamases, including ESBLs, AmpC enzymes and carbapenemases, represents an important contemporary unmet medical need (6).

Boronate-based β-lactamase inhibitors have recently been approved (vaborbactam) or are in late-stage clinical development (taniborbactam) (7) (ClinicalTrials.gov Identifier: NCT03840148) for use in combination with approved β-lactams to treat antimicrobial-resistant infections. VNRX-7145 (VNRX-5236 etzadroxil) is a novel, orally bioavailable, cyclic boronate β-lactamase inhibitor in development for use in combination with ceftibuten (an oral third-generation cephalosporin) as a potential oral treatment for complicated urinary tract infections caused by serine β-lactamase-producing *Enterobacterales*. *In vivo*, the prodrug VNRX-7145 undergoes rapid and extensive biotransformation to the active β-lactamase inhibitor VNRX-5236 (8–10). VNRX-5236 covalently and reversibly binds and inhibits the active site serine of Ambler class A, C, and D β-lactamases (8–10). The combination of VNRX-5236 and ceftibuten has shown potent inhibitory activity against multidrug-resistant (MDR) *Enterobacterales* expressing Ambler class A, C and D β-lactamases including those that hydrolyze carbapenems such as KPCs and OXAs (11, 12). VNRX-5236 alone does not demonstrate antibacterial activity.

Ceftibuten/VNRX-7145 is currently in phase 1 clinical trials that involve first-in-human dose-ranging studies to evaluate the safety and pharmacokinetics of escalating doses of VNRX-7145 (ClinicalTrials.gov identifier: NCT04243863). In this study ceftibuten/VNRX-5236 and 16 comparators were tested against 1,066 MDR urinary isolates of *Enterobacterales* chosen from a 2014–2016 global culture collection. The MDR phenotype of each isolate tested was preselected to include nonsusceptibility to amoxicillin-clavulanate, resistance to levofloxacin, and nonsusceptibility to one or more additional oral and parenteral agents from other structural categories where agents have potential for use in treating patients with complicated or uncomplicated urinary tract infections (13). The objective of this study was to determine the ability of VNRX-5236 to restore the activity of ceftibuten against this challenge set of antimicrobial-resistant isolates.

## RESULTS

**FIG 1:**
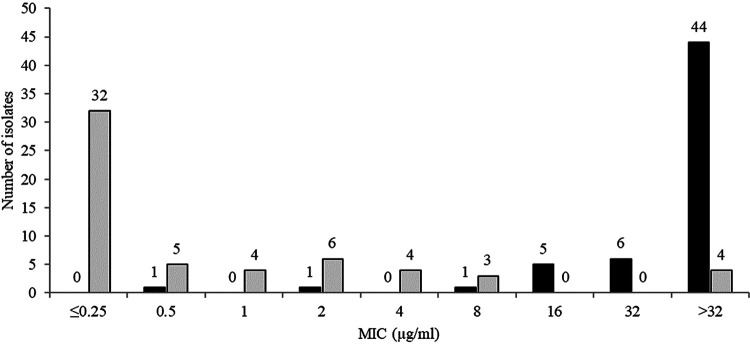
MIC distributions for ceftibuten (black) and ceftibuten/VNRX-5236 (gray) for 1,066 urinary isolates of *Enterobacterales* preselected to possess a MDR phenotype that included nonsusceptibility to amoxicillin-clavulanate and resistance to levofloxacin.

The *in vitro* activities of ceftibuten/VNRX-5236 and comparators against all 1,066 urinary isolates of *Enterobacterales* tested are shown in Table 1. The MIC_90_ for ceftibuten in combination with a fixed 4 μg/ml of VNRX-5236 was 2 μg/ml, which was at least 32-fold lower than for ceftibuten alone (MIC_90_, >32 μg/ml). At a ceftibuten concentration of 1 μg/ml (EUCAST susceptible breakpoint for ceftibuten) (14), ceftibuten/VNRX-5236 and ceftibuten alone inhibited 89.1% (950/1,066) and 25.5% (272/1,066) of isolates, respectively (Fig. 1). Ceftibuten/VNRX-5236 at 2 μg/ml inhibited 91.7% (978/1,066) of isolates compared to only 33.4% of isolates (356/1,066) for ceftibuten alone at 2 μg/ml. Ceftibuten/VNRX-5236 at 4 μg/ml inhibited 93.1% (992/1,066) of isolates compared with only 44.5% of isolates (474/1,066) for ceftibuten alone at 4 μg/ml. Using CLSI/EUCAST susceptible breakpoint criteria, all other oral agents tested demonstrated percent susceptible rates that ranged from 5.8 to 11.0% (cefuroxime) to 42.3 to 54.0% (nitrofurantoin). MIC_90_s for cefepime-taniborbactam, ceftazidime-avibactam, and meropenem-vaborbactam were 1–2 μg/ml. Ceftolozane-tazobactam (MIC_90_ >8 μg/ml) and piperacillin-tazobactam (MIC_90_ >64 μg/ml) were less active than other β-lactam-β-lactamase inhibitor combinations, with only 55.9 and 23.0% (EUCAST) or 32.7% (CLSI) of isolates, respectively, categorized as susceptible.

**TABLE 1:** *In vitro* activity of ceftibuten/VNRX-5236 and comparator agents against 1,066 urinary isolates of *Enterobacterales* preselected to possess a MDR phenotype that included nonsusceptibility to amoxicillin-clavulanate and resistance to levofloxacin

Antimicrobial agent	MIC_50_^*e*^	MIC_90_	MIC range	MIC interpretation: CLSI			MIC interpretation: EUCAST	
				% susceptible	% intermediate	% resistant	% susceptible	% resistant
Ceftibuten/VNRX-5236^*a*^	≤0.25	2	≤0.25–>32	NA^*b*^	NA	NA	NA	NA
Ceftibuten	8	>32	0.12–>32	58.3	13.0	28.8	25.5	74.5
Cefepime-taniborbactam^*c*^	≤0.25	2	≤0.25–>32	NA	NA	NA	NA	NA
Cefepime	32	>32	≤0.25–>32	40.3^*d*^	NA	59.7	20.8	72.0
Amoxicillin-clavulanate	32	>64	16–>64	0	46.1	53.9	0	100
Cefazolin	>16	>16	≤0.5->16	0.9	3.2	95.9	0	95.9
Cefixime	>4	>4	≤0.12->4	13.5	3.8	82.7	13.5	86.5
Ceftazidime–avibactam	0.25	2	≤0.03–>32	97.2	NA	2.8	97.2	2.8
Ceftolozane-tazobactam	2	>8	≤0.25–>8	55.9	8.3	35.8	55.9	44.1
Ceftriaxone	>64	>64	≤1–>64	3.5	16.8	79.7	3.5	79.7
Cefuroxime	>16	>16	≤1–>16	5.8	7.6	86.6	11.0	89.0
Fosfomycin	64	>128	≤1–>128	NA	NA	NA	43.2	56.8
Levofloxacin	>4	>4	4–>4	0	0	100	0	100
Meropenem-vaborbactam	0.03	1	0.015–>8	95.2	1.1	3.7	96.3	3.7
Nitrofurantoin	64	>64	4–>64	42.3	11.7	46.0	54.0	46.0
Piperacillin-tazobactam	64	>64	≤0.5–>64	32.7	22.0	45.3	23.0	67.3
Trimethoprim-sulfamethoxazole	>32	>32	≤2–>32	19.9	NA	80.1	19.9	78.1

a VNRX-5236 was tested at a fixed concentration of 4 μg/ml in combination with doubling dilutions of ceftibuten.

b NA, not applicable.

c Taniborbactam was tested at a fixed concentration of 4 μg/ml in combination with doubling dilutions of cefepime.

d Percentage determined using the cefepime susceptible-dose dependent breakpoint. CLSI does not define an intermediate MIC breakpoint for cefepime tested against *Enterobacterales*.

e All MIC values in the table are μg/ml.

Table 2 summarizes the relationship between ceftibuten MICs alone and MICs for ceftibuten in combination with VNRX-5236 (at a fixed concentration of 4 μg/ml). Of the 621 isolates with ceftibuten MICs ≤8 μg/ml, only two isolates tested with an MIC >1 μg/ml for ceftibuten/VNRX-5236 (both isolates had an MIC of 2 μg/ml). In the remaining subset of 445 isolates with ceftibuten MICs of 16 to >32 μg/ml, higher ceftibuten MICs were associated with higher ceftibuten/VNRX-5236 MICs, however, VNRX-5236 potentiated ceftibuten activity overall, restoring ceftibuten MICs to ≤1 μg/ml for 74.4% (331/445) of isolates, to ≤2 μg/ml for 80.2% (357/445) of isolates, and to ≤4 μg/ml for 83.4% (371/445) of isolates. Figure S1 in the supplemental material depicts the same data set showing the relationship between ceftibuten MICs and ceftibuten/VNRX-5236 MICs in graphical form.

**TABLE 2:** *In vitro* activity of ceftibuten/VNRX-5236 at ceftibuten concentrations of 1, 2, and 4 μg/ml against 1,066 urinary isolates of *Enterobacterales* preselected to possess a MDR phenotype that included nonsusceptibility to amoxicillin-clavulanate and resistance to levofloxacin stratified by ceftibuten MIC

Cefibuten MIC, μg/ml (no.)	Ceftibuten/VNRX-5236^*a*^					
	MIC_50_	MIC_90_	MIC range	Percentage of isolates inhibited by VNRX-5236 at a concentration of 4 μg/ml in combination with ceftibuten at a concentration of:		
				1 μg/ml	2 μg/ml	4 μg/ml
≤0.25 (173)	≤0.25	≤0.25	≤0.25–0.5	100	100	100
0.5 (50)	≤0.25	≤0.25	≤0.25	100	100	100
1 (49)	≤0.25	≤0.25	≤0.25–2	98.0	100	100
2 (84)	≤0.25	≤0.25	≤0.25–0.5	100	100	100
4 (118)	≤0.25	≤0.25	≤0.25–1	100	100	100
8 (147)	≤0.25	≤0.25	≤0.25–2	99.3	100	100
16 (138)	≤0.25	0.5	≤0.25–16	94.2	97.8	98.6
32 (112)	≤0.25	2	≤0.25–>32	84.8	91.1	95.5
>32 (195)	1	>32	≤0.25–>32	54.4	61.5	65.6

a VNRX-5236 was tested at a fixed concentration of 4 μg/ml in combination with doubling-dilutions of ceftibuten. All MIC values in the table are μg/ml.

Table 3 presents the *in vitro* activity of ceftibuten/VNRX-5236 against isolates with defined, not susceptible phenotypes (the not susceptible phenotypes listed are in addition to the basal amoxicillin-clavulanate not susceptible and levofloxacin-resistant phenotype characteristic of all isolates tested). Ceftibuten/VNRX-5236 at concentrations of ≤1, ≤2, and ≤4 μg/ml inhibited 77–90%, 82–92%, and 84–93% of isolates that were not susceptible (defined by CLSI and EUCAST breakpoint criteria) to cefepime, cefazolin, cefixime, ceftolozane-tazobactam, ceftriaxone, cefuroxime, fosfomycin, nitrofurantoin, piperacillin-tazobactam, and trimethoprim-sulfamethoxazole as part of their MDR phenotype.

**TABLE 3:** *In vitro* activity of ceftibuten/VNRX-5236 at ceftibuten concentrations of 1, 2, and 4 μg/ml against urinary isolates of *Enterobacterales* preselected to possess a MDR phenotype that included nonsusceptibility to amoxicillin-clavulanate and resistance to levofloxacin; isolates are stratified by phenotypic nonsusceptibility to agents other than amoxicillin-clavulanate and levofloxacin that were associated with MDR phenotypes

Isolates not susceptible to (no.)		Ceftibuten/VNRX-5236^*a*^					
	MIC breakpoint criteria				Percentage of isolates inhibited by VNRX-5236 at a concentration of 4 μg/ml in combination with ceftibuten at a concentration of:		
		MIC_50_	MIC_90_	MIC range	1 μg/ml	2 μg/ml	4 μg/ml
Cefepime (722)	CLSI^*b*^	≤0.25	4	≤0.25–>32	87.0	89.6	90.9
Cefepime (844)	EUCAST	≤0.25	4	≤0.25–>32	87.2	89.8	91.4
Cefazolin (1,056)	CLSI	≤0.25	2	≤0.25–>32	89.0	91.7	93.0
Cefazolin (1,066)	EUCAST	≤0.25	2	≤0.25–>32	89.1	91.7	93.1
Cefixime (922)	CLSI and EUCAST	≤0.25	2	≤0.25–>32	87.4	90.5	92.0
Ceftazidime-avibactam (30)	CLSI and EUCAST	>32	>32	2–>32	0	3.3	6.7
Ceftolozane-tazobactam (470)	CLSI and EUCAST	≤0.25	32	≤0.25–>32	76.8	81.5	84.3
Ceftriaxone (1,029)	CLSI and EUCAST	≤0.25	2	≤0.25–>32	88.8	91.4	92.8
Cefuroxime (1,004)	CLSI	≤0.25	2	≤0.25–>32	88.4	91.2	92.6
Cefuroxime (949)	EUCAST	≤0.25	2	≤0.25–>32	87.8	90.7	92.2
Fosfomycin (606)	EUCAST	≤0.25	4	≤0.25–>32	85.6	89.4	91.4
Meropenem-vaborbactam (51)	CLSI	8	>32	≤0.25–>32	35.3	45.1	45.1
Meropenem-vaborbactam (39)	EUCAST	16	>32	≤0.25–>32	30.8	41.0	41.0
Nitrofurantoin (616)	CLSI	≤0.25	4	≤0.25–>32	84.7	89.1	91.2
Nitrofurantoin (491)	EUCAST	≤0.25	4	≤0.25–>32	82.5	87.8	90.4
Piperacillin-tazobactam (717)	CLSI	≤0.25	>32	≤0.25–>32	84.0	87.7	89.7
Piperacillin-tazobactam (821)	EUCAST	≤0.25	4	≤0.25–>32	85.9	89.3	91.0
Trimethoprim-sulfamethoxazole (854)	CLSI and EUCAST	≤0.25	2	≤0.25–>32	88.5	91.1	92.4

a VNRX-5236 was tested at a fixed concentration of 4 μg/ml in combination with doubling dilutions of ceftibuten. Specific analysis of data for cefibuten, amoxicillin-clavulanate, and levofloxacin were excluded from this table. A similar analysis of ceftibuten data is presented in Table 2. All MIC values in the table are μg/ml.

b The isolates tested do not include isolates with cefepime susceptible-dose dependent (SDD) MICs.

**FIG 2:**
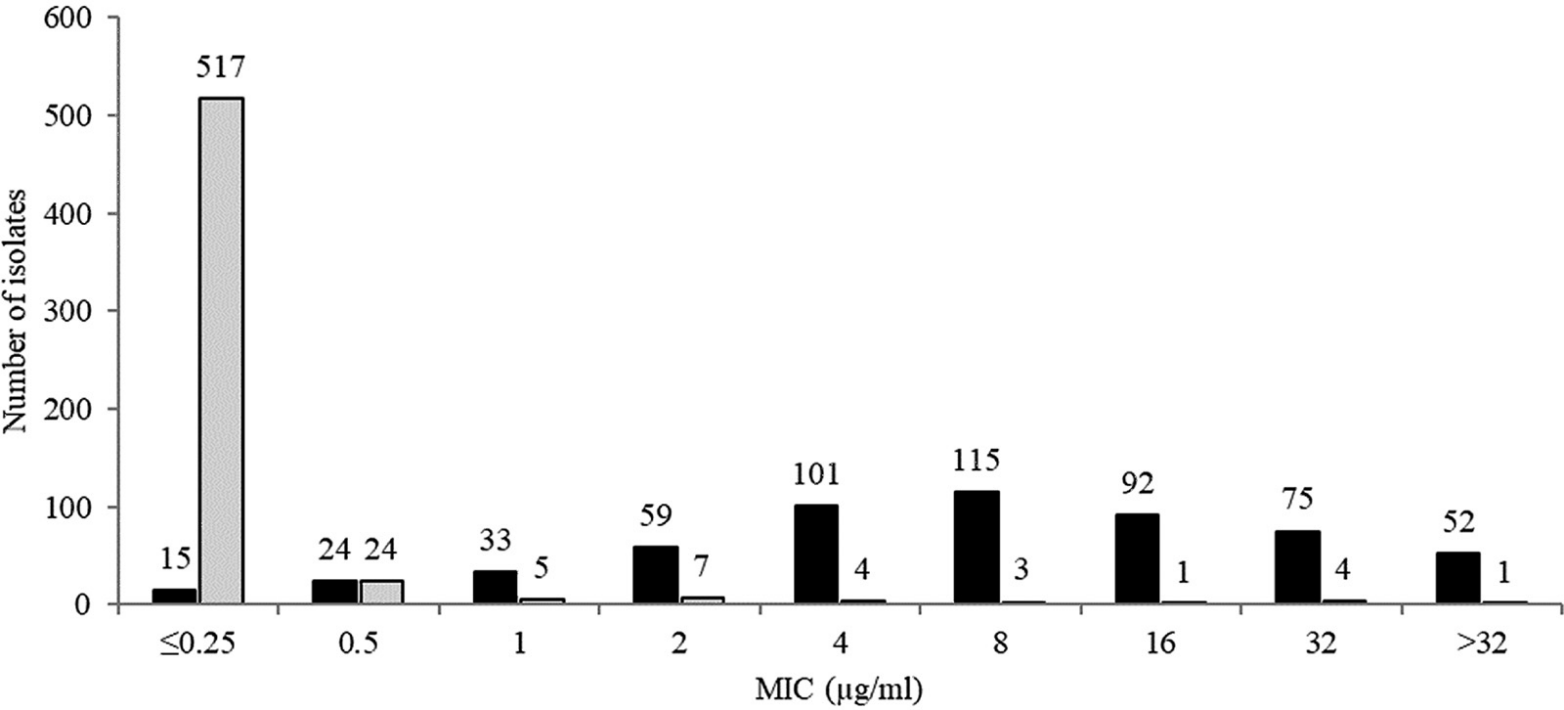
MIC distributions for ceftibuten (black) and ceftibuten/VNRX-5236 (gray) for 566 urinary isolates of ESBL-positive *Enterobacterales* (excluded isolates simultaneously carrying an acquired AmpC, a serine carbapenemase, or a metallo-β-lactamase).

Table 4 summarizes the β-lactamase genotypic content of the 1,066 isolates tested. The *in vitro* activities of ceftibuten/VNRX-5236 and comparators against 566 ESBL-positive *Enterobacterales* (excluding isolates carrying an acquired AmpC, a serine carbapenemase, or metallo-β-lactamases) are shown in Table 5. The MIC_90_ for ceftibuten/VNRX-5236 was ≤0.25 μg/ml compared to 32 μg/ml for ceftibuten alone. At a ceftibuten concentration of 1 μg/ml, ceftibuten/VNRX-5236 and ceftibuten alone inhibited 96.5% (546/566) and 12.7% (72/566) of isolates, respectively (Table 4, Fig. 2). Ceftibuten/VNRX-5236 at a concentration of 2 μg/ml inhibited 97.7% (553/566) of isolates compared to 23.1% (131/566) for ceftibuten alone at 2 μg/ml. Ceftibuten/VNRX-5236 at a concentration of 4 μg/ml inhibited 98.4% (557/566) of isolates compared to 41.0% (232/566) for ceftibuten alone at 4 μg/ml. Ceftibuten/VNRX-5236 (MIC_90_ ≤0.25 μg/ml) was more potent than ceftazidime-avibactam (MIC_90_ 1 μg/ml) and of similar potency to meropenem-vaborbactam (MIC_90_ 0.06 μg/ml) against ESBL-positive isolates. In contrast, only 30.7% of ESBL-positive isolates were susceptible to piperacillin-tazobactam (MIC_90_ >64 μg/ml) and only 59.5% were susceptible to ceftolozane-tazobactam (MIC_90_ >8 μg/ml).

**TABLE 4:** *In vitro* activity of ceftibuten/VNRX-5236 and comparator agents against 1,066 urinary isolates of *Enterobacterales* preselected to possess a MDR phenotype that included nonsusceptibility to amoxicillin-clavulanate and resistance to levofloxacin based on β-lactamase content

Genotype (inclusions; exclusions)			Percentage of isolates inhibited by VNRX-5236 at a concentrationof 4 μg/ml in combination with ceftibuten at a concentration of:		
	No. of isolates	Prevalence, as % of 1,066 isolates	1 μg/ml	2 μg/ml	4 μg/ml
All isolates	1,066	100	89.1	91.7	93.1
OSBL only/unknown	296	27.8	92.6	94.9	96.6
ESBL (no acquired AmpC, no serine carbapenemase, no metallo-β-lactamase)	566	53.1	96.5	97.7	98.4
CTX-M	546	51.2	96.5	97.6	98.4
CTX-M + SHV	9	0.8	100	100	100
SHV	10	0.9	90.0	100	100
TEM	1	0.1	100	100	100
Acquired AmpC (+/- ESBL; no serine carbapenemase, no metallo-β-lactamase)	58	5.4	70.7	81.0	87.9
ACC	1	0.1	100	100	100
CMY	30	2.8	86.7	86.7	86.7
CMY + DHA	1	0.1	100	100	100
DHA	26	2.4	50.0	73.1	88.5
Serine carbapenemase (+/- ESBL, +/- acquired AmpC; no metallo-β-lactamase)	116	10.9	75.9	81.9	81.9
KPC	60	5.6	81.7	91.7	91.7
KPC, no acquired AmpC	58	5.4	81.0	91.4	91.4
KPC + CMY	2	0.2	100	100	100
OXA-48/-48-like	55	5.2	69.1	70.9	70.9
OXA-48/OXA-48-like, no acquired AmpC	40	3.8	92.5	95.0	95.0
OXA-48/OXA-48-like + CMY	13	1.2	7.7	7.7	7.7
OXA-48/OXA-48-like + DHA	2	0.2	0	0	0
OXA-48/OXA-48-like + KPC	1	0.1	100	100	100
Metallo-β-lactamase	30	2.8	3.3	6.7	10.0
IMP	2	0.2	0	0	0
NDM	19	1.8	0	0	0
VIM	9	0.8	11.1	22.2	33.3

**TABLE 5:** *In vitro* activity of ceftibuten/VNRX-5236 and comparator agents against 566 urinary isolates of ESBL-positive *Enterobacterales*^*a*^

Antimicrobial agent	MIC_50_	MIC_90_	MIC range	MIC interpretation – CLSI			MIC interpretation – EUCAST	
				% susceptible	% intermediate	% resistant	% susceptible	% resistant
Ceftibuten/VNRX-5236^*b*^	≤0.25	≤0.25	≤0.25–>32	NA^*c*^	NA	NA	NA	NA
Ceftibuten	8	32	0.12–>32	61.3	16.3	22.4	12.7	87.3
Cefepime-taniborbactam^*d*^	≤0.25	1	≤0.25–32	NA	NA	NA	NA	NA
Cefepime	>32	>32	0.5–>32	8.5^*e*^	NA	91.5	0.9	94.5
Amoxicillin-clavulanate	16	32	16–>64	0	69.1	30.9	0	100
Cefazolin	>16	>16	≤0.5–>16	0.4	0.5	99.1	0	99.1
Cefixime	>4	>4	≤0.12–>4	1.1	0.2	98.7	1.1	98.9
Ceftazidime-avibactam	0.25	1	≤0.03–8	100	NA	0	100	0
Ceftolozane-tazobactam	2	>8	≤0.25–>8	59.5	9.9	30.6	59.5	40.5
Ceftriaxone	>64	>64	≤1–>64	0	0.7	99.3	0	99.3
Cefuroxime	>16	>16	≤1–>16	0.2	1.2	98.6	0.4	99.6
Fosfomycin	64	>128	≤1–>128	NA	NA	NA	47.5	52.5
Levofloxacin	>4	>4	4–>4	0	0	100	0	100
Meropenem-vaborbactam	0.03	0.06	0.015–4	100	0	0	100	0
Nitrofurantoin	64	>64	4–>64	47.1	12.5	40.3	59.7	40.3
Piperacillin-tazobactam	64	>64	≤0.5–>64	30.7	27.7	41.5	18.7	81.3
Trimethoprim-sulfamethoxazole	>32	>32	≤2–>32	17.5	NA	82.5	17.4	80.6

a ESBL-positive isolates excluded isolates simultaneously carrying an acquired AmpC, a serine carbapenemase, or a metallo-β-lactamase. The 566 isolates were comprised of 283 E. coli, 266 K. pneumoniae, 14 Proteus mirabilis, and 3 K. oxytoca. All MIC values in the table are μg/ml.

b VNRX-5236 was tested at a fixed concentration of 4 μg/ml in combination with doubling dilutions of ceftibuten.

c NA, not applicable.

d Taniborbactam was tested at a fixed concentration of 4 μg/ml in combination with doubling dilutions of cefepime.

e Percentage determined using the cefepime susceptible-dose dependent breakpoint. CLSI does not define an intermediate MIC breakpoint for cefepime tested against *Enterobacterales*.

**FIG 3:**
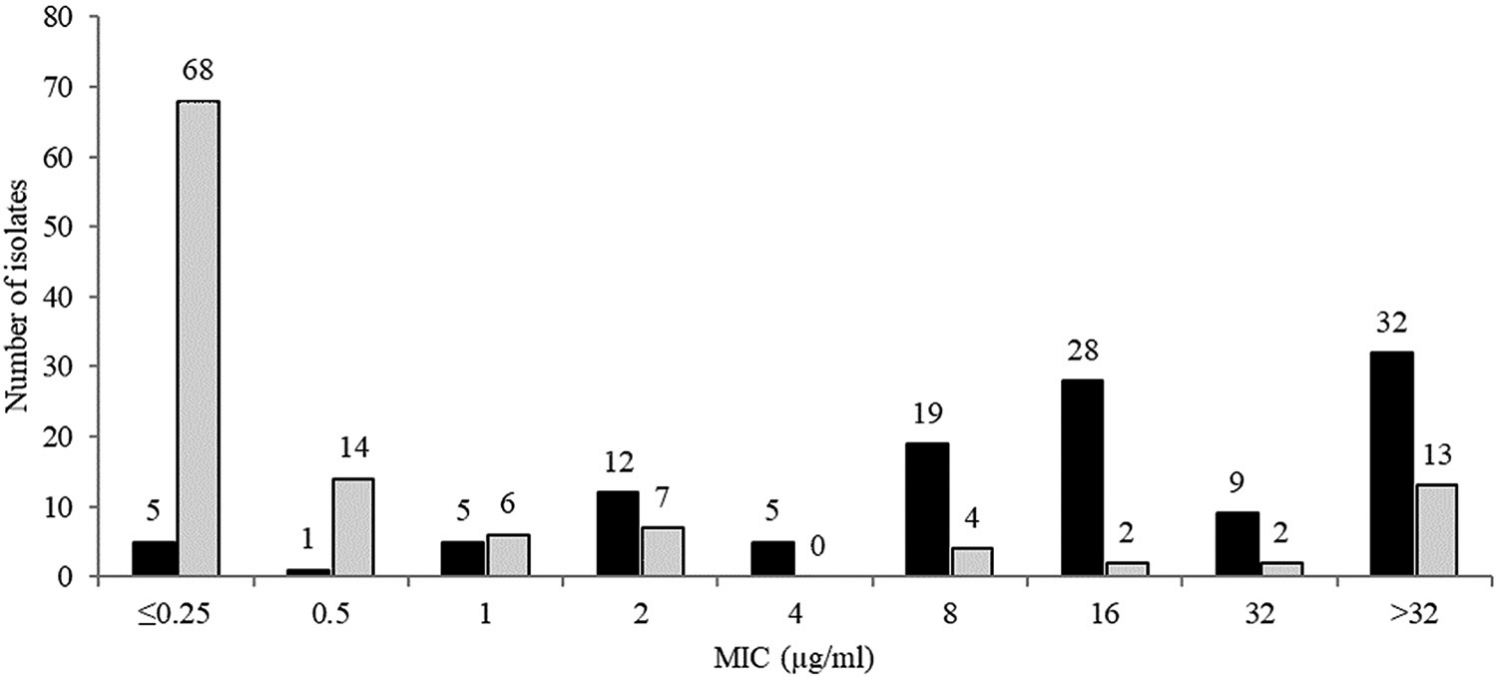
MIC distributions for ceftibuten (black) and ceftibuten/VNRX-5236 (gray) for 116 urinary isolates of serine carbapenemase-positive *Enterobacterales* (included isolates carrying KPC and/or OXA together with or without ESBLs or acquired AmpC but excluded isolates carrying metallo-β-lactamases).

The *in vitro* activities of ceftibuten/VNRX-5236 and comparators against 116 serine carbapenemase-positive *Enterobacterales* (included isolates carrying KPC and/or OXA together with or without ESBLs or acquired AmpC but excluded isolates carrying metallo-β-lactamases) are summarized in Table 6. The MIC_50_ for ceftibuten/VNRX-5236 was ≤0.25 μg/ml compared to 16 μg/ml for ceftibuten alone; MIC_90_s were >32 μg/ml for both ceftibuten/VNRX-5236 and ceftibuten alone. At a concentration of 1 μg/ml, 75.9% (88/116) and 9.5% (11/116) of isolates, respectively, were inhibited by ceftibuten/VNRX-5236 and ceftibuten alone (Table 4, Fig. 3). Ceftibuten/VNRX-5236 at a concentration of 2 μg/ml inhibited 81.9% (95/116) of isolates compared to 19.8% (23/116) for ceftibuten at 2 μg/ml. Ceftibuten/VNRX-5236 at a concentration of 4 μg/ml inhibited 81.9% (95/116) of isolates compared to 24.1% (28/116) for ceftibuten at 4 μg/ml. Ceftibuten/VNRX-5236 at concentrations of 1, 2, and 4 μg/ml inhibited 81.7, 91.7, and 91.7% of KPC-positive isolates and 92.5, 95.0, and 95.0% of OXA-48-like-positive isolates that did not carry an acquired AmpC enzyme (Table 4). Isolates carrying both an OXA-48-like enzyme and an acquired AmpC were generally not inhibited by ceftibuten/VNRX-5236. Ceftibuten/VNRX-5236 was less active than ceftazidime-avibactam (MIC_90_ 2 μg/ml) and cefepime-taniborbactam (MIC_90_ 4 μg/ml) against serine carbapenemase-positive isolates.

**TABLE 6:** *In vitro* activity of ceftibuten/VNRX-5236 and comparator agents against 116 urinary isolates of serine carbapenemase-positive *Enterobacterales*^*a*^

Antimicrobial agent	MIC_50_	MIC_90_	MIC range	MIC interpretation: CLSI			MIC interpretation: EUCAST	
				% susceptible	% intermediate	% resistant	% susceptible	% resistant
Ceftibuten/VNRX-5236^*b*^	≤0.25	>32	≤0.25–>32	NA^*c*^	NA	NA	NA	NA
Cefitibuten	16	>32	0.12–>32	40.5	24.1	35.3	9.5	90.5
Cefepime-taniborbactam^*d*^	1	4	≤0.25–32	NA	NA	NA	NA	NA
Cefepime	>32	>32	≤0.25–>32	9.5^*e*^	NA	90.5	1.7	95.7
Amoxicillin-clavulanate	>64	>64	16–>64	0	1.7	98.3	0	100
Cefazolin	>16	>16	2–>16	0.9	0	99.1	0	99.1
Cefixime	>4	>4	0.12–>4	2.6	2.6	94.8	2.6	97.4
Ceftazidime-avibactam	1	2	0.06–16	99.1	NA	0.9	99.1	0.9
Ceftolozane-tazobactam	>8	>8	≤0.25–>8	2.6	0.9	96.6	2.6	97.4
Ceftriaxone	>64	>64	≤1–>64	0.9	2.6	96.6	0.9	96.6
Cefuroxime	>16	>16	≤1–>16	0.9	0.9	98.3	1.7	98.3
Fosfomycin	>128	>128	4–>128	NA	NA	NA	18.1	81.9
Levofloxacin	>4	>4	>4	0	0	100	0	100
Meropenem-vaborbactam	1	>8	0.03–>8	75.0	6.9	18.1	81.9	18.1
Nitrofurantoin	>64	>64	4–>64	20.7	6.0	73.3	26.7	73.3
Piperacillin-tazobactam	>64	>64	16–>64	0.9	0.9	98.3	0	100
Trimethoprim-sulfamethoxazole	>32	>32	≤2–>32	15.5	NA	84.5	15.5	82.7

a Serine carbapenemase-positive isolates included isolates carrying KPC and/or OXA together with or without ESBLs or acquired AmpC but excluded isolates carrying metallo-β-lactamases. The 116 isolates were comprised of 91 K. pneumoniae, 19 Escherichia coli, 3 Klebsiella oxytoca, 2 Citrobacter freundii, and 1 Enterobacter cloacae. All MIC values in the table are μg/ml.

b VNRX-5236 was tested at a fixed concentration of 4 μg/ml in combination with doubling dilutions of ceftibuten.

c NA, not applicable.

d Taniborbactam was tested at a fixed concentration of 4 μg/ml in combination with doubling dilutions of cefepime.

e Percentage determined using the cefepime susceptible-dose dependent breakpoint. CLSI does not define an intermediate MIC breakpoint for cefepime tested against *Enterobacterales*.

**FIG 4:**
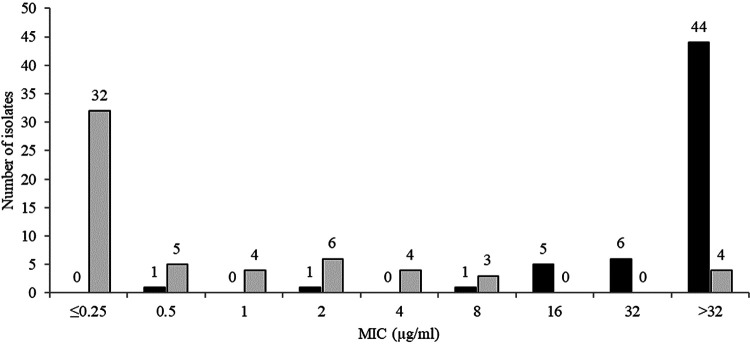
MIC distributions for ceftibuten (black) and ceftibuten/VNRX-5236 (gray) for 58 urinary isolates of acquired AmpC-positive *Enterobacterales* (included isolates with or without ESBLs but excluded isolates carrying serine carbapenemases and metallo-β-lactamases).

The *in vitro* activities of ceftibuten/VNRX-5236 and comparators against 58 acquired AmpC-positive *Enterobacterales* (included isolates with or without ESBLs but excluded isolates carrying serine carbapenemases or metallo-β-lactamases) are summarized in Table 7. The MIC_90_ for ceftibuten/VNRX-5236 against acquired AmpC-positive isolates was 8 μg/ml compared to >32 μg/ml for ceftibuten alone. Ceftibuten/VNRX-5236 at concentrations of 1, 2, and 4 μg/ml inhibited 70.7, 81.0, and 87.9 of acquired AmpC-positive isolates (Table 4, Fig. 4). Ceftibuten/VNRX-5236 was less active than meropenem-vaborbactam (MIC_90_ 0.06 μg/ml), ceftazidime-avibactam (MIC_90_ 1 μg/ml), and cefepime-taniborbactam (MIC_90_ 1 μg/ml) against acquired AmpC-positive isolates.

**TABLE 7:** *In vitro* activity of ceftibuten/VNRX-5236 and comparator agents against 58 urinary isolates of acquired AmpC-positive *Enterobacterales*^*a*^

Antimicrobial agent	MIC_50_	MIC_90_	MIC range	MIC interpretation: CLSI			MIC interpretation: EUCAST	
				% susceptible	% intermediate	% resistant	% susceptible	% resistant
Ceftibuten/VNRX-5236^*b*^	≤0.25	8	≤0.25–>32	NA^*c*^	NA	NA	NA	NA
Cefitibuten	>32	>32	0.5–>32	5.2	8.6	86.2	1.7	98.3
Cefepime-taniborbactam^*d*^	≤0.25	1	≤0.25–16	NA	NA	NA	NA	NA
Cefepime	4	>32	≤0.25–>32	62.1^*e*^	NA	37.9	37.9	46.6
Amoxicillin-clavulanate	32	64	32–>64	0	0	100	60.3	39.7
Cefazolin	>16	>16	>16	0	0	100	0	100
Cefixime	>4	>4	>4	0	0	100	0	100
Ceftazidime-avibactam	0.25	1	0.06–8	100	NA	0	100	0
Ceftolozane-tazobactam	4	>8	0.5–>8	46.6	20.7	32.8	46.6	53.4
Ceftriaxone	32	>64	≤1–>64	3.4	15.5	81.0	3.4	81.0
Cefuroxime	>16	>16	4–>16	1.7	1.7	96.6	1.7	98.3
Fosfomycin	64	>128	4–>128	NA	NA	NA	44.8	55.2
Levofloxacin	>4	>4	4–>4	0	0	100	0	100
Meropenem-vaborbactam	0.03	0.06	0.03–8	98.3	1.7	0	100	0
Nitrofurantoin	64	>64	8–>64	46.6	5.2	48.3	51.7	48.3
Piperacillin-tazobactam	64	>64	1–>64	29.3	27.6	43.1	22.4	77.6
Trimethoprim-sulfamethoxazole	>32	>32	≤2–>32	17.2	NA	82.8	17.2	79.3

a Acquired AmpC-positive isolates included isolates with or without ESBLs but excluded isolates carrying serine carbapenemases and metallo-β-lactamases. The 58 KPC isolates were comprised of 26 K. pneumoniae, 26 Escherichia coli, and 6 Proteus mirabilis. All MIC values in the table are μg/ml.

b VNRX-5236 was tested at a fixed concentration of 4 μg/ml in combination with doubling dilutions of ceftibuten.

c NA, not applicable.

d Taniborbactam was tested at a fixed concentration of 4 μg/ml in combination with doubling dilutions of cefepime.

e Percentage determined using the cefepime susceptible-dose dependent breakpoint. CLSI does not define an intermediate MIC breakpoint for cefepime tested against *Enterobacterales*.

For the 30 metallo-β-lactamase-positive isolates in the culture collection, the MIC_50_ and MIC_90_ for ceftibuten/VNRX-5236 were both >32 μg/ml (IHMA, data on file). Similarly, MIC_90_s (and the percentage of susceptible isolates) for ceftazidime-avibactam, meropenem-vaborbactam, and piperacillin-tazobactam were elevated at >32 μg/ml (3.3%), >8 μg/ml (40.0%), and >64 μg/ml (0%), respectively, for the 30 metallo-β-lactamase-positive isolates (IHMA, data on file). For comparative purposes, cefepime-taniborbactam (MIC_90_, >32 μg/ml) inhibited 66.7% of the metallo-β-lactamase-positive isolates at its provisional susceptible MIC breakpoint of ≤8 μg/ml (IHMA, data on file).

## DISCUSSION

Ceftibuten in combination with VNRX-7145 (VNRX-5236 etzadroxil) is under development as an oral treatment for complicated urinary tract infections caused by serine β-lactamase-producing *Enterobacterales*, including isolates carrying ESBLs and carbapenemases (10). In the current study we challenged ceftibuten/VNRX-5236 using a recent collection of 1,066 urinary isolates of *Enterobacterales* preselected to possess a MDR phenotype that included nonsusceptibility to amoxicillin-clavulanate and resistance to levofloxacin. We preselected isolates that were MDR and possessed resistance to potential empirical first-line agents (amoxicillin-clavulanate and levofloxacin) used in the treatment of patients with complicated urinary tract infections, including acute pyelonephritis, and included phenotypes identified as CDC and WHO priority pathogens (i.e., carbapenem-resistant and/or third-generation cephalosporin-resistant *Enterobacterales*) (2, 3). CDC and WHO priority pathogens necessitate the development of new agents, particularly oral agents, for outpatient treatment to reduce duration of hospitalization or avoid admission altogether (2, 3). The current study determined that the addition of VNRX-5236 (4 μg/ml) to ceftibuten restored the activity of ceftibuten in many resistant isolates.

Increasing resistance among agents commonly prescribed to treat urinary tract infections (trimethoprim-sulfamethoxazole, fluoroquinolones, oral β-lactams including amoxicillin-clavulanate) and for agents with pharmacokinetic (nitrofurantoin) and spectrum limitations (nitrofurantoin, fosfomycin) indicate that new oral agents are urgently needed to address the inadequacies of current agents (6, 15). In the current study, ceftibuten/VNRX-5236 (MIC_90_ 2 μg/ml) was significantly more potent *in vitro* than fosfomycin (MIC_90_ >128 μg/ml), nitrofurantoin (MIC_90_ >64 μg/ml), and trimethoprim-sulfamethoxazole (MIC_90_ >32 μg/ml), and it inhibited a majority of isolates that were not susceptible to each of these agents in addition to their nonsusceptibility to amoxicillin-clavulanate and resistance to levofloxacin. Ceftibuten/VNRX-5236 may therefore also have promise in patients where resistant pathogens are suspected or in some patients with complicated infection where hospital avoidance and oral therapy is reasonable.

Ceftibuten/VNRX-5236 demonstrated an *in vitro* potency that is similar to established and newer parenteral therapies. MIC_90_s for ceftibuten/VNRX-5236, ceftazidime-avibactam, and meropenem-vaborbactam were all 1–2 μg/ml. In contrast, ceftolozane-tazobactam was less active than other β-lactam-β-lactamase inhibitor combinations, with an MIC_90_ of >8 μg/ml and only 55.9% of isolates categorized as susceptible. Ceftibuten/VNRX-5236, therefore, may also have potential as a step-down oral agent from a broad-spectrum empirical or directed parenteral agent (e.g., ceftazidime-avibactam or meropenem-vaborbactam) where *Enterobacterales* producing serine β-lactamases, including carbapenemases, is confirmed or highly suspected. Against MDR *Enterobacterales*, ceftibuten/VNRX-5236 has been reported to demonstrate similar potency *in vitro* to carbapenems and current β-lactam/β-lactamase inhibitors such as meropenem, meropenem-vaborbactam, and ceftazidime-avibactam (16). These results are consistent with those in the current study and suggest that ceftibuten/VNRX-5236 should be further developed as a potential carbapenem-sparing oral agent to treat patients with complicated urinary tract infections due to MDR *Enterobacterales*.

In the current study, ceftibuten/VNRX-5236 demonstrated potent *in vitro* activity against ESBL-producing isolates of *Enterobacterales* that did not co-produce a class C or a class D β-lactamase (Table 4). VNRX-5236 restored ceftibuten MICs to ≤1 μg/ml for 86.7% of isolates expressing CMY, whether alone or in combination with an ESBL, consistent with its spectrum of inhibition against this enzyme family (8, 10). However, DHA-producing *Enterobacterales*, whether alone or in combination with an ESBL (but without serine carbapenemases or metallo-β-lactamases), generated higher ceftibuten/VNRX-5236 MICs than did isolates without these enzymes present. We also observed that although ceftibuten/VNRX-5236 is active against OXA-48/OXA-48-like producers in isolation or in isolates also producing an ESBL, MICs are elevated against isolates co-producing OXA-48/OXA-48-like and CMY or OXA-48/OXA-48-like and DHA (again, without serine carbapenemases or metallo-β-lactamases). While the epidemiology of β-lactam resistance may change across time and geographies, the proportion of isolates expressing DHA alone (with or without ESBLs) or OXA-48/OXA-48-like and an acquired AmpC enzyme (with or without ESBLs) was limited to 2.4 and 1.4% of isolates in the current challenge set, respectively.

In the current study, ceftibuten activity was not restored by VNRX-5236 for metallo-β-lactamase-producing isolates. This observation is consistent with the spectrum of inhibitory activity of VNRX-5236 as determined in biochemical assays (8, 10) and is similar to the activities of other tested agents in the current study including the carbapenem-boronate β-lactamase inhibitor combination, meropenem-vaborbactam. Currently no approved β-lactam-β-lactamase inhibitor combination covers metallo-β-lactamase-producing isolates. There are only two β-lactam-β-lactamase inhibitor combinations in development that possess a spectrum that covers metallo-β-lactamases; cefepime-taniborbactam and aztreonam-avibactam. Cefepime-taniborbactam was evaluated in the present study; taniborbactam restored the activity of cefepime to ≤8 μg/ml for 20 of the 28 isolates that expressed NDM or VIM and any combination of ESBL, acquired AmpC and/or serine carbapenemases.

Previously published data describing the *in vitro* activity of ceftibuten/VNRX-5236 against which to compare our data are currently limited. Chatwin et al. reported MIC_90_s of 0.25, 1, 1, and 1 μg/ml for ceftibuten/VNRX-5236 (4 μg/ml) tested against ESBL- (*n *= 25), KPC- (*n *= 25), OXA-48- (*n *= 25) and class C-carrying (*n *= 25) isolates of *Enterobacterales* (11). Mendes et al. studied the activity of ceftibuten/VNRX-5236 (4 μg/ml) against a challenge set of 205 isolates of *Enterobacterales* that included ESBL- (*n* = 50), KPC- (*n* = 50), OXA-48-like- (*n* = 52) and class C-positive (*n* = 53) isolates and reported MIC_90_s of 0.12, 0.5, 1, and 1 μg/ml, respectively (12). The application of EUCAST susceptible breakpoints for ceftibuten (≤1 μg/ml) to their data showed 98.0% of ESBL-positive isolates, 92.0% of KPC-positive isolates, 94.0% of OXA-48-like-positive isolates, and 94.3% of acquired AmpC-positive isolates were inhibited by ceftibuten/VNRX-5236 (12). Overall, MIC results for ceftibuten/VNRX-5236 (MIC_50_ 0.12 μg/ml, MIC_90_ 1 μg/ml) were 256-fold lower than for ceftibuten alone (MIC_50_ 32 μg/ml, MIC_90_ 256 μg/ml) against all *Enterobacterales* tested and 2- to 4-fold lower than for ceftazidime-avibactam (MIC_50_ 0.5 μg/ml, MIC_90_ 2 μg/ml) (12). The slightly higher ceftibuten/VNRX-5236 MIC_90_ values and corresponding lower percentages of isolates inhibited at 1 and 2 μg/ml in the present study might be explained by the basal MDR phenotype, including nonsusceptibility to amoxicillin-clavulanate and resistance to levofloxacin, which potentially suggests that the isolates reported here may have greater levels of β-lactamase production and/or accumulated multiple additional resistance mechanisms (presumably entry- and/or efflux-related) compared with those isolates reported by other investigators.

Uehara et al. demonstrated that over-expression of an ESBL (CTX-M-15) or KPC (KPC-2, KPC-3) in isogenic strains of E. coli did not significantly increase ceftibuten-VNRX-5236 (4 μg/ml) MICs while the over-expression of an AmpC β-lactamase (P99, CMY-42) increased MICs from 0.25 μg/ml (control) to 4 μg/ml (17). This observation suggests that the overexpression of certain β-lactamases may account for the relatively reduced activity of ceftibuten/VNRX-5236 observed in the current study compared with earlier studies, as described above, with the caveat that analysis of gene expression would be needed to definitively identify the reason for the discordance.

There is currently discordance between CLSI and EUCAST MIC breakpoints for ceftibuten. CLSI investigational MIC breakpoints for ceftibuten tested against *Enterobacterales* are susceptible, ≤8 μg/ml; intermediate, 16 μg/ml; and resistant, ≥32 μg/ml; these breakpoints are for testing and reporting of urinary tract isolates of *Enterobacterales* only (18). EUCAST MIC breakpoints for ceftibuten tested against *Enterobacterales* are: susceptible, ≤1 μg/ml and resistant, >1 μg/ml; these breakpoints apply to infections originating in the urinary tract (14). Initial pharmacokinetic/pharmacodynamic data for VNRX-5236 suggests that the free area under the concentration-time curve to MIC ratio (*f*AUC_0-24_/MIC) is the parameter that best correlates with *in vivo* activity of VNRX-5236 (i.e., dosing frequency of VNRX-5236 does not impact the extent of VNRX-5236 potentiation of ceftibuten activity) and that based upon a hypothetical clinical dose of 300 mg of ceftibuten given every 8 h that the susceptible MIC breakpoint for ceftibuten/VNRX-5236 may be in the range of 1 to 2 μg/ml (i.e., closer to the current EUCAST breakpoint than the current CLSI breakpoint) (19). Further nonclinical and clinical studies are required to justify doses of the combination for registrational clinical studies and to support eventual breakpoint decisions.

The. First, each isolate of *Enterobacterales* tested was preselected to possess a MDR phenotype that current study has at least three important limitations included nonsusceptibility to amoxicillin-clavulanate and resistance to levofloxacin. Such an isolate collection, enriched for resistant phenotypes, invalidates comparisons with prevalence-based studies. Second, isolates in the current study were not characterized for non-β-lactamase-mediated resistance mechanisms (e.g., porin mutation/expression and efflux pump expression), which are known to affect the activity of cephalosporins, including ceftibuten, and β-lactam-β-lactamase inhibitor combinations (20). Third, no data are included regarding isolate background, including clinical syndrome and underlying host comorbidities.

Based on the data generated in the current study, ceftibuten/VNRX-5236 appears to have potential as an oral treatment option for complicated urinary tract infections caused by serine β-lactamase-expressing *Enterobacterales* (ESBL, KPC, OXA-48/OXA-48-like) for which there are currently few oral treatment options available. Ceftibuten/VNRX-5236 also exhibited potent *in vitro* activity against isolates that were not susceptible to current, frequently prescribed oral (fosfomycin, nitrofurantoin, and trimethoprim-sulfamethoxazole) and parenteral (ceftriaxone, piperacillin-tazobactam, and ceftolozane-tazobactam) agents. Further clinical development of ceftibuten-VNRX-7145 is warranted.

## MATERIALS AND METHODS

### Bacterial isolates.

Community- and hospital-associated urinary tract infection isolates of *Enterobacterales* (*n *= 1,066) were chosen from a 2014–2016 global culture collection maintained by IHMA (Schaumburg, IL). Each isolate was preselected to possess an MDR phenotype as defined by the criteria of Magiorakos et al. (13). The MDR phenotype included nonsusceptibility to amoxicillin-clavulanate (MIC ≥16 μg/ml), resistance to levofloxacin (MIC ≥4 μg/ml), as well as nonsusceptibility to one or more additional oral and parenteral agents from other antimicrobial categories (Table 1) where agents have potential for use in treating patients with complicated or uncomplicated urinary tract infections (13). Determination of nonsusceptible phenotypes was based on 2021 CLSI (18) and EUCAST (14) MIC breakpoint criteria. The 1,066 isolates tested originated from the following regions (*n*, percentage of total): Africa (16, 1.5%), Asia (116, 10.9%), Europe (506, 47.5%), Latin America (246, 23.1%), Middle East (75, 7.0%), North America (62, 5.8%), and South Pacific (45, 4.2%). Speciation of the isolates tested is summarized in Table S1 in the supplemental material.

The 1,066 isolates were previously tested for the presence of genes encoding β-lactamases using published multiplex PCR assays, followed by full-gene DNA sequencing (21, 22). The isolates were screened for genes encoding ESBLs (CTX-M, GES, PER, SHV, TEM, VEB), serine carbapenemases (GES, KPC, OXA), acquired AmpC β-lactamases (ACC, ACT, CMY, DHA, FOX, MIR, MOX), and metallo-β-lactamases (GIM, IMP, NDM, SPM, VIM). One or more ESBL genes (661 CTX-M [542 CTX-M-15, 32 CTX-M-2, 31 CTX-M-14, 56 others], 37 SHV [30 SHV-12, 7 others], 1 TEM [TEM-226], 1 VEB-1) were present in 634 isolates. A KPC (42 KPC-2; 19 KPC-3) was identified in 61 isolates, an OXA-48-like carbapenemase (34 OXA-48, 14 OXA-181, 7 OXA-232, 5 others) was identified in 60 isolates, an acquired AmpC β-lactamase (49 CMY, 29 DHA, 1 ACC, 1 ACT) was identified in 80 isolates, and a metallo-β-lactamase (19 NDM, 9 VIM, 2 IMP) was identified in 30 isolates (IHMA, data on file).

### Antimicrobial susceptibility testing.

MICs were determined using the CLSI reference broth microdilution method (23). Broth microdilution panels were prepared at IHMA using cation-adjusted Mueller-Hinton broth (CAMHB) (Becton, Dickinson, Sparks, MD) and stored at −80°C until the day of testing. CAMHB with TES (TREK Diagnostic Systems, Independence, OH) was used for inoculum preparation. Tryptic soy agar (TSA) plates containing 5% sheep blood (Liofilchem, Waltham, MA) were used to subculture isolates.

MICs were interpreted using CLSI (18) and EUCAST (14) breakpoints. In instances where CLSI or EUCAST publish multiple MIC breakpoint criteria for the same agent, the following criteria were used for amoxicillin-clavulanate, the EUCAST uncomplicated urinary tract infection only breakpoints were used (susceptible, ≤32 μg/ml; resistant, >32 μg/ml); for cefazolin, the CLSI parenteral breakpoints were used (susceptible, ≤2 μg/ml; intermediate 4 μg/ml; resistant, ≥8 μg/ml); for cefuroxime, the CLSI oral breakpoints (susceptible, ≤4 μg/ml; intermediate 8–16 μg/ml; resistant, ≥32 μg/ml) and EUCAST oral breakpoints (susceptible, ≤8 μg/ml; resistant, >8 μg/ml) were used; for fosfomycin, the EUCAST parenteral breakpoints (susceptible, ≤32 μg/ml; resistant, >32 μg/ml) were used. CLSI publishes investigational MIC breakpoints for ceftibuten (susceptible, ≤8 μg/ml; intermediate 16 μg/ml; resistant, ≥32 μg/ml) for testing and reporting of urinary tract isolates only (18). EUCAST publishes MIC breakpoints for ceftibuten (susceptible, ≤1 μg/ml; resistant, >1 μg/ml) for infections originating from the urinary tract (14).

VNRX-5236 and taniborbactam were provided by Venatorx Pharmaceuticals, Inc. (Malvern, PA). Other antimicrobial agents were purchased from commercial sources. VNRX-5236 and taniborbactam were dissolved in DMSO to make initial solutions of 5,120 μg/ml; these solutions were diluted 1:10 in sterile water to create 512 μg/ml stock solutions. MICs for ceftibuten/VNRX-5236 and cefepime-taniborbactam were determined at a fixed concentration of 4 μg/ml for VNRX-5236 and taniborbactam.

MICs for VNRX-5236 and taniborbactam combinations were read as the first microdilution panel well with no visible growth following 16 to 20 h of incubation at 35°C in ambient air. Quality control testing was performed each day clinical isolates were tested using E. coli ATCC 25922, P. aeruginosa ATCC 27853, and K. pneumoniae ATCC 700603 (18, 23). Consensus reference quality control ranges and strains for broth microdilution testing of ceftibuten/VNRX-5236 have been determined (CLSI 2021 Winter AST Plenary 05A QCWG Report Draft 4, https://clsi.org/meetings/ast-file-resources/) but remain to be published. The E. coli ATCC 25922 quality control range for ceftibuten was used as the quality control range for ceftibuten/VNRX-5236.
