# Supplementary material for: In Vitro Activity of Ceftibuten/VNRX-5236 against Urinary Tract Infection Isolates of Antimicrobial-Resistant Enterobacterales
Source: Antimicrob Agents Chemother. 2022 Jan 18;66(1):e01304-21. doi: 10.1128/AAC.01304-21 (PMC8765315; doi:10.1128/AAC.01304-21)
Supplement: Supplemental file 1 — Supplemental table and figure. Download AAC.01304-21-s0001.pdf, PDF file, 0.10 MB [file aac.01304-21-s0001.pdf]

SUPPLEMENTAL TABLE 1 Total isolate counts by species of Enterobacterales and by year of collection

Species of Enterobacterales	Total isolates (% of total isolates)	Year collected		
		2014	2015	2016
Citrobacter braakii	3 (0.3)		2	1
Citrobacter freundii	38 (3.6)		14	24
Citrobacter koseri	1 (0.1)		1	
Enterobacter aerogenes	7 (0.7)		5	2
Enterobacter asburiae	1 (0.1)			1
Enterobacter cloacae	49 (4.6)		30	19
Escherichia coli	429 (40.2)		193	236
Klebsiella oxytoca	10 (0.9)		6	4
Klebsiella pneumoniae	435 (40.8)	1	221	213
Morganella morganii	22 (2.1)		6	16
Proteus mirabilis	26 (2.4)		16	10
Proteus vulgaris	1 (0.1)		1	
Providencia rettgeri	9 (0.8)		2	7
Providencia stuartii	33 (3.1)		8	25
Serratia marcescens	2 (0.2)			2
Total	1,066 (100)	1	505	560

SUPPLEMENTAL FIG 1 Scatterplot of ceftibuten and ceftibuten/VNRX-5236 MICs for 1,066 urinary isolates of Enterobacterales pre-selected to possess a MDR phenotype that included non-susceptibility to amoxicillin-clavulanate and resistance to levofloxacin.

Ceftibuten/VNRX-5236 MIC (μg/ml)	≤0.25	0.5	1	2	4	8	16	32	>32
>32								1	39
32									8
16							1	2	8
8							1	2	12
4							1	5	8
2			1			1	5	7	14
1					1	2	3	3	12
0.5	2		2	4	3	7	11	9	20
≤0.25	171	50	46	80	114	137	116	83	74

Ceftibuten MIC (μg/ml)
